# Widely targeted metabolomics reveal the distribution of primary and secondary metabolites in pomegranate fruit

**DOI:** 10.1002/fsn3.4264

**Published:** 2024-06-26

**Authors:** Lina Chen, Ruitao Liu, Juanli Zhu, Luwei Wang, Haoxian Li, Junhao Liu, Zhenhua Lu

**Affiliations:** ^1^ Zhengzhou Fruit Research Institute Chinese Academy of Agricultural Sciences Zhengzhou China; ^2^ Zhongyuan Research Center Chinese Academy of Agricultural Sciences Xinxiang China; ^3^ National Nanfan Research Institute Chinese Academy of Agricultural Sciences Sanya China; ^4^ Chuxiong Yunguo Agriculture Technology Research Institute Chinese Academy of Agricultural Sciences Yunnan China; ^5^ National Key Laboratory for Germplasm Innovation & Utilization of Horticultural Crops Zhengzhou Fruit Research Institute Zhengzhou China

**Keywords:** metabolites profiling, nutrition, pomegranate, taste

## Abstract

The pomegranate fruit is valued for its nutritional and medicinal properties, and the composition and content of primary and secondary metabolites are the main factors impacting its nutritional and medicinal properties. However, a deep understanding of metabolites in different parts of fruit is still lacking. Here, the peel, aril, and seed of mature pomegranate fruits were analyzed separately to compare metabolic component differences using UPLC/MS–MS. A total of 858 metabolites belonging to 11 classes were identified, of which flavonoids, such as delphinidin‐3‐O‐glucoside and cyanidin‐3‐O‐glucoside; tannins, such as ellagic acid and punicalin; and terpenoids, such as corosolic acid and madasiatic acid, were upregulated in the peel. Lipids, such as punicic acid, methyl linolenate, and linoleic acid; alkaloids, such as indole and choline; nucleotides and derivatives mainly including 2‐deoxyribose‐1‐phosphate and 9‐(arabinosyl)‐hypoxanthine, were upregulated in seeds. Phenolic acids, such as 1‐O‐galloyl‐4,6‐(S)‐HHDP‐β‐D‐glucose and 1,7‐di‐O‐galloyl‐D‐sedoheptulose, and flavonoids, such as cyanidin‐3‐O‐glucoside, cyanidin‐3‐O‐(2″‐O‐xylosyl) galactoside, and delphinidin‐3‐O‐glucoside, were upregulated in aril. The flavone and flavonol biosynthesis (ko00944) pathways were significantly enriched between the peel and seed, as were the anthocyanin biosynthesis (ko00944) pathways between the aril and seed and the flavonoid biosynthesis (ko00941) pathways between the peel and aril. Additionally, functional antioxidants, such as 10,16‐dihydroxypalmitic acid, 3‐O‐methylellagic acid, and 3,3’‐O‐dimethylellagic acid, were first identified in pomegranate fruits. Our results revealed the composition and abundance of primary and secondary metabolites in pomegranate fruit, which can lay the foundation for further elucidation of its nutritional and medicinal properties.

## Graphical abstract

A total of 858 metabolites belonging to 11 classes were identified in pomegranate peel, seed, and aril, of which 647 metabolites showed statistically significant differences between the groups. Flavonoids were mainly upregulated in peel and aril; as were phenolic acids and amino acids in aril; tannins and terpenoids in peel; and lipids and alkaloids in seed. Some functional antioxidants, such as 10,16‐dihydroxypalmitic acid, 3‐O‐methylellagic acid, and 3,3′‐O‐dimethylellagic acid, were first identified in pomegranate fruits.

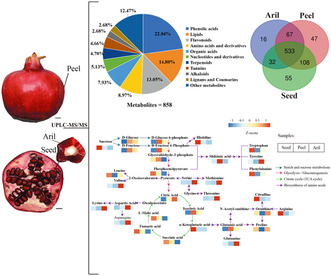

## 1 INTRODUCTION

Pomegranate (*Punica granatum* L., Lythraceae) is an economically important perennial fruit tree with a wide global geographical distribution. It is valued for its nutritional, medicinal, and ornamental properties (Gosset‐Erard et al., 2021; Sharma et al., 2017). Its fruit has been renowned for its health benefits in many diseases, such as diarrhea (Larrosa et al., 2010), respiratory pathologies (Viuda‐Martos et al., 2010), androgen‐independent prostate cancer (Vicinanza et al., 2013), gastric cancer (Cheshomi et al., 2022), and memory in adults (Yang et al., 2020).

Pomegranate fruits mainly comprise three parts: peels, multi‐seeds, and membranous walls (Holland et al., 2009). Seeds consist of seed coats and embryos, of which the seed coat mainly includes outer and inner seed coats. The outer seed coat (also named aril) is a special layer of juice cells, and the inner seed coat, together with the embryo, is called the seed (Qin et al., 2020). Arils, peels, and seeds are the main edible or processed parts of pomegranate fruit (Bar‐Ya'akov et al., 2019). The peels are processed into powder for medical (Khwairakpam et al., 2018) or bioactive additions (Kumar et al., 2022); the seeds are processed into oil (Liu et al., 2009); and the arils are used freshly or for the preparation of juice, wine, jam, or vinegar (Turrini et al., 2015). Most of the taste, nutritional, and medicinal properties of pomegranate fruit are mainly attributed to the composition and content of primary and secondary metabolites. Therefore, it is important to clarify the composition and content of primary and secondary metabolites in pomegranates.

Several primary and secondary metabolites, such as organic acids, sugars, lipids, phenolic compounds, and flavonoids, have been identified in pomegranate peel, arils, and seeds in previous studies. Primary metabolites, mainly 10 types of sugars in the aril and peel (Dafny‐Yalin et al., 2010), 10 organic acids in the aril or peel (Dafny‐Yalin et al., 2010; Hasnaoui et al., 2011; Melgarejo et al., 2000; Poyrazolua et al., 2002), 22 amino acids and derivatives in the aril, peel, or seed (Elfalleh et al., 2011; Li et al., 2017; Rowayshed et al., 2013), and 66 types of lipids mainly in seeds were detected based on high‐performance liquid chromatography (HPLC) or amino acid analysis. Secondary metabolites, such as 6 major anthocyanins (Borochov‐Neori et al., 2011; Zhao et al., 2013) in the peel and arils, 22 phenolic compounds in the peel (Abid et al., 2017), 16 hydrolyzable tannins in the peel (Yaritz et al., 2022), and 26 flavonoids in the peel (Yaritz et al., 2022), were identified via LC–MS/MS and HPLC.

However, limited by the detection method, many functional metabolites have still not been identified, and an in‐depth investigation of the fruit metabolites in different tissues (peel, aril, and seed) is lacking. It is necessary for enhancing the utilization of the chemical and functional components of pomegranate. Widely targeted metabolomics, which features high throughput and is widespread, is an effective method for detecting the biochemical and metabolic composition of an organism based on UPLC/MS–MS (Chen et al., 2009). It has been used to determine the key taste and nutritional components of fruits in many species, such as loquat (Zou et al., 2020), pear (Zheng et al., 2022), wampee (Yin et al., 2022), and apricot (Cui et al., 2022).

Thus, we further evaluated the components and relative amounts in the peel, aril, and seed tissues of mature pomegranate fruits using UPLC–MS/MS. Our results provide comprehensive data on the metabolite distribution in pomegranate fruit, which can improve our understanding of the metabolite composition and relative content in the peel, aril, and seed, laying the foundation for the development of pomegranate functional substances and quality trait‐related studies.

## 2 MATERIALS AND METHODS

### 2.1 Plant materials

**FIGURE 1:**
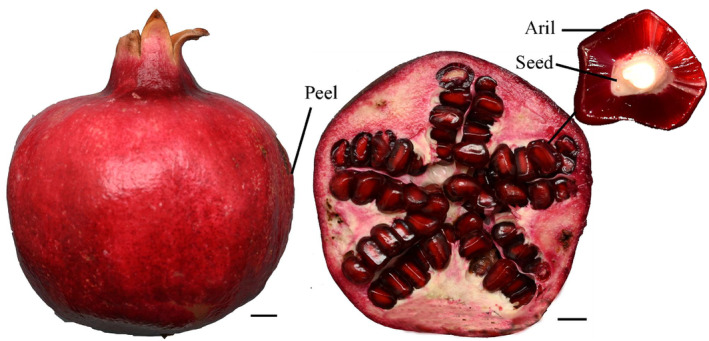
Morphology of peel, aril, and seed in mature fruits from “Zhongshiliu No. 4.”

The peels, seeds, and arils of the pomegranate cultivar “Zhongshiliu No. 4” were used in this study (Figure 1). Samples were collected from 7‐year‐old trees growing at the National Horticultural Germplasm Resource Center (Zhengzhou, China) located at 34°45′16.19″ N, 113°40′39.17″ E, which were cultured under standard horticultural practices.

### 2.2 Sample preparation and extraction

We separated the peels, seeds, and arils of three mature fruits obtained from three trees into one biological sample. Three biological samples (≥9 fruits in total) were generated for each part. Samples were stored at −80°C after harvesting for metabolite extraction. The powder was vortexed for 10 s to mix with the samples for further processing. After mixing, 50 mg of the sample was placed into a 2‐mL centrifuge tube, 1200 μL of 70% methanol internal standard extract was added, scrolled for 3 min, and centrifuged (12,000 r/min, 4°C) for 10 min. Finally, a microporous membrane was used to filter the supernatant before storing it in a sample flask for UPLC‐MS/MS analysis.

### 2.3 UPLC‐MS/MS analysis

The sample extracts were analyzed using an Agilent SB‐C18 column (1.8 μm, 2.1 mm × 100 mm) on a UPLC‐MS/MS system (UPLC, SHIMADZU Nexera X2, MS/MS, Applied Biosystems 4500 QTRAP). The conditions mainly included flow velocity: 0.35 mL/min, column oven: 40°C, and injection volume: 4 μL. ESI‐triple quadrupole linear ion trap (QTRAP)‐MS was used to analyze the effluent.

### 2.4 Determination of organic acid content

According to previous methods (Feng et al., 2022), we performed HPLC to determine the contents of oxalic acid, quininic acid, malic acid, shikimic acid, and citric acid among the arils of two acidless and three acid varieties. Chromatographic column: Xselect HSS T3 (4.6 mm × 250 mm, 5 μm), mobile phase: 0.02 mol/L diammonium phosphate (phosphoric acid adjusted pH 2.4), detection wavelength: 210 nm, flow rate: 1.0 mL/min, and column temperature: 30°C.

### 2.5 Data processing

#### 2.5.1 Qualitative and quantitative analyses of metabolites

Mass spectral data were analyzed using Analyst 1.6.3 (Chen et al., 2013). Based on the Metware database, messages from the MS were obtained for qualitative analysis. The MRM system was selected to ensure the accuracy of the quantitative analysis. MultiaQuant software was used for the integration and calibration of the chromatographic peaks. Each chromatographic peak corresponds to the relative amount of each substance (Fraga et al., 2010).

#### 2.5.2 Pearson correlation coefficient analysis

The coefficient of variation (CV), the ratio of the standard deviation to the mean of the original data, was calculated to reflect the discrete degrees of the different samples. Principal component analysis (PCA) and Pearson's correlation coefficient (PCC) were used to evaluate the overall differences between samples. The prcomp function in R was used for unsupervised PCA (Chen et al., 2009; Eriksson et al., 2006; Thévenot et al., 2015). The metabolites of different samples were clustered as heat maps using dendrograms. Hierarchical cluster analysis (HCA) and PCC were performed using R packages. For HCA, the color spectrum was visualized by normalizing the metabolite signal intensities.

#### 2.5.3 Differential metabolite selection

We performed OPLS‐DA to analyze the significantly regulated metabolites between groups (variable importance in projection [VIP] ≥ 1, log_2_FC (fold change) ≥ 1). The MetaboAnalystR package in R was used to generate VIP values (Chong & Xia, 2018). An overfitting test was performed (200 permutations).

#### 2.5.4 KEGG annotation and enrichment analysis

The KEGG Compound database was used to annotate metabolites. The annotated metabolites were mapped to the KEGG Pathway database (Kanehisa & Goto, 2000). The significance of the pathways was determined using the *p*‐values of the hypergeometric test.

## 3 RESULTS AND DISCUSSION

### 3.1 Compositions of primary and secondary metabolites in pomegranate peel, aril, and seed

Several primary and secondary metabolites have been extracted from pomegranate fruits in previous reports, which have mainly focused on specific classes of metabolites, such as sugars (Dafny‐Yalin et al., 2010), organic acids (Mphahlele et al., 2016), amino acids (Li et al., 2017), and phenolic acids (Kalaycıoğlu & Erim, 2017) in aril; lipids and fatty acids in seed (Melgarejo & Artes, 2000); and ellagitannins, flavonoids, and anthocyanin in peel (Hasnaoui et al., 2014). However, comprehensive information on the composition and abundance of metabolites in pomegranate peel, aril, and seed is lacking.

**FIGURE 2:**
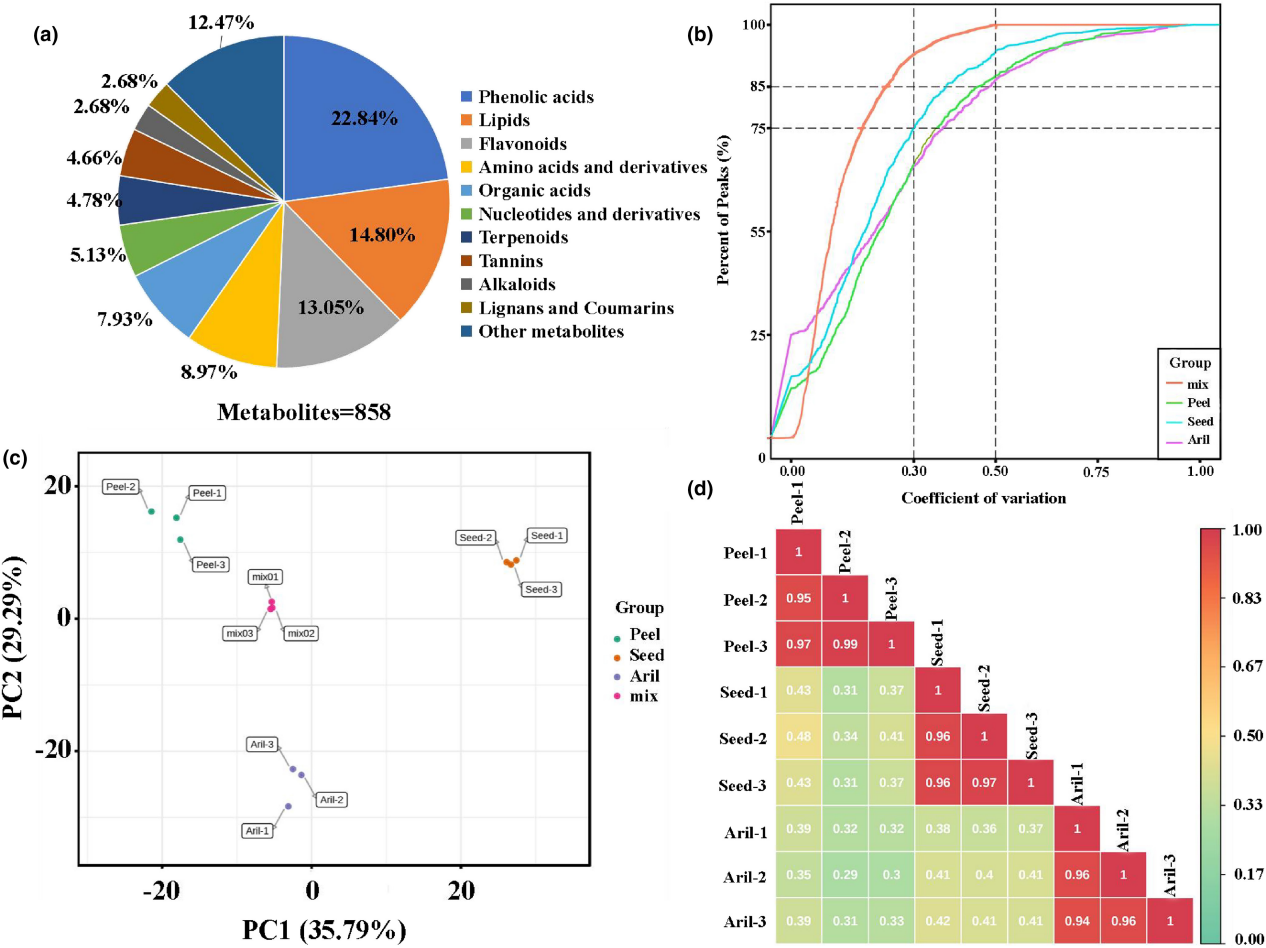
Multivariate analysis of the identified metabolites. (a) Classification of the 858 metabolites of pomegranate fruits. (b) CV of different groups and QC samples; mix represents QC samples. (c) The PCA score plot of different groups and QC samples. (d) The heatmap of PCC among different groups.

In this study, we performed targeted metabolite profiling of the peel, aril, and seed tissues of “Zhongshiliu No.4” using UPLC/MS–MS. A total of 858 metabolites belonging to 11 classes were identified, including many that may contribute to taste, nutritional, and medicinal values (Table S1). The metabolites included 196 phenolic acids (22.84%), 127 lipids (14.80%), 112 flavonoids (13.05%), 77 amino acids and derivatives (8.97%), 68 organic acids (7.93%), 44 nucleotides and derivatives (5.13%), 41 terpenoids (4.78%), 40 tannins (4.66%), 23 alkaloids (2.68%), 23 lignans and coumarins (2.68%), and 107 other metabolites (12.47%) (Figure 2a, Table S1). Other metabolites included saccharides, alcohols, and vitamins. Many important metabolites with high nutritional values, such as D‐threonic acid, manninotriose, sorbitol‐6‐phosphate, γ‐glutamyltyrosine, and 10,16‐dihydroxypalmitic acid, were first identified in our results.

### 3.2 Differential metabolites among pomegranate peel, aril, and seed

The ratio of metabolites whose CV ≤0.5 was greater than 85% (Figure 2b) showed the stability of the experimental data. The two principal components explained 65.08% of the total variance, representing high cohesion within groups and sufficient discrete cohesion among peel, seed, and aril tissues (Figure 2c). PC1 and PC2 contributed to 35.79% and 29.29% of the variance, respectively (Figure 2c). Moreover, the PCC between different samples, including the same group and different tissues, showed high cohesion within groups and discrete degrees among different tissues (Figure 2d).

**FIGURE 3:**
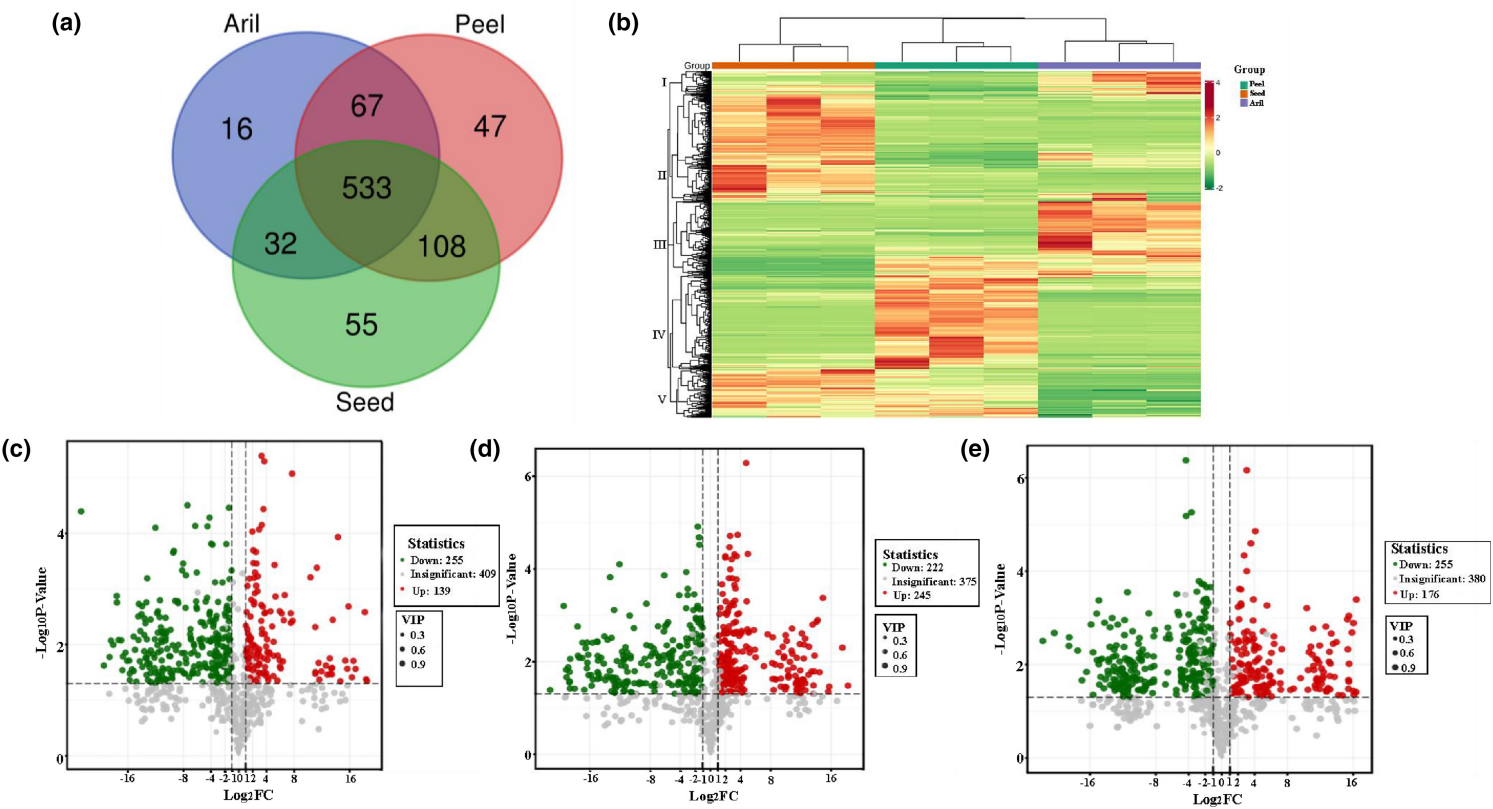
Unique and shared metabolites and differential metabolites analysis among different groups. (a) Venn diagram illustrating the overlapping and specific metabolites for three comparison groups. (b) HCA map of metabolites. Each sample is represented by a column; the green column represents group peel; the orange column represents group seed; and the purple column represents group aril. Each metabolite is displayed in a row. Red shows relatively high metabolite abundance, and green shows relatively low abundance. (c) OPLS‐DA score plots between peel and aril groups. (d) Score plots generated from OPLS‐DA in peel vs. seed groups. (e) The seed vs. aril groups.

A total of 755, 728, and 648 metabolites were identified in peel, seed, and aril tissues, respectively (Table S1). Among these metabolites, 533 compounds were identified across all groups, 108 metabolites were specifically detected in the peel and seeds, 67 in the peel and aril, 32 in the aril and seeds, 47 in the peel, 16 in the aril, and 55 in the seeds (Figure 3a, Table S2). The metabolites were clustered into five main groups using HCA (Figure 3b). Cluster I metabolites showed the highest content in seeds and arils, cluster II showed the highest level in seeds, cluster III exhibited the highest level in arils, cluster IV mainly accumulated in peels, and cluster V showed the highest content in peels and seeds (Figure 3b).

The metabolites whose VIP ≥1, FC ≥2, and *p*_value ≤.05 between groups were selected as differential metabolites. A total of 647 metabolites showed statistically significant differences between the groups. Between peel and aril, the number of significantly different metabolites was 394 (Figure 3c), whereas there were 467 significantly different metabolites in peel vs. seed (Figure 3d) and 431 in seed versus aril (Figure 3e).

**FIGURE 4:**
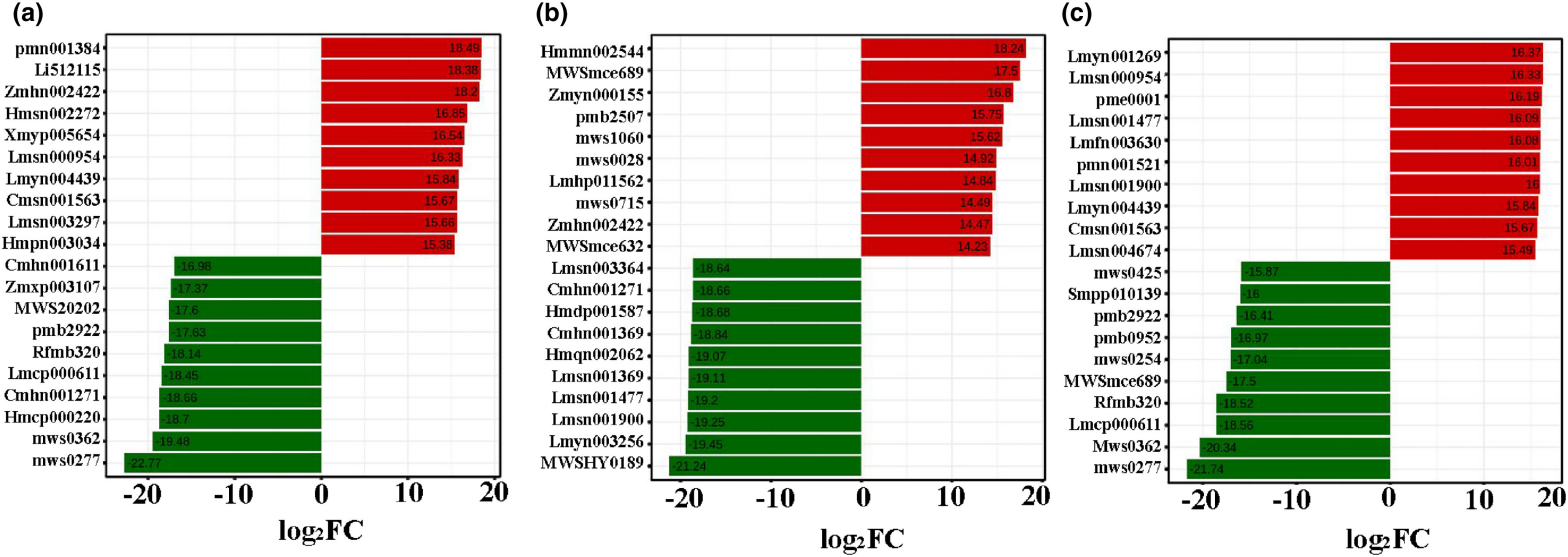
Bar chart of the top 10 upregulated and downregulated metabolites between different groups. (a) Between peel and aril. (b) Between peel and seed. (c) Between seed and aril. The X‐axis is the log2FC of metabolites between different groups, while the Y‐axis is the index of differential metabolites.

Of the 394 differential metabolites between peel and aril, 255 metabolites mainly belonging to tannins, terpenoids, and flavonoids classes were upregulated in peel (Figure 4a, Table S3), and 139 metabolites mainly comprising phenolic acids and amino acids members were upregulated in aril (Figure 4a, Table S3).

Between peel and seed, a total of 245 metabolites, mainly belonging to lipids, nucleotides and derivatives, and phenolic acid classes were upregulated in the seed and 222 metabolites, mainly including tannins and flavonoids members, were upregulated in peel (Figure 4b, Table S4).

Moreover, of the 431 significantly different metabolites in seeds vs. arils, 255 metabolites, mainly including lipids and alkaloids members, were upregulated in seeds, while 176 metabolites mainly belonging to the flavonoids and phenolic acid classes were upregulated in arils (Figure 4c, Table S5).

**FIGURE 5:**
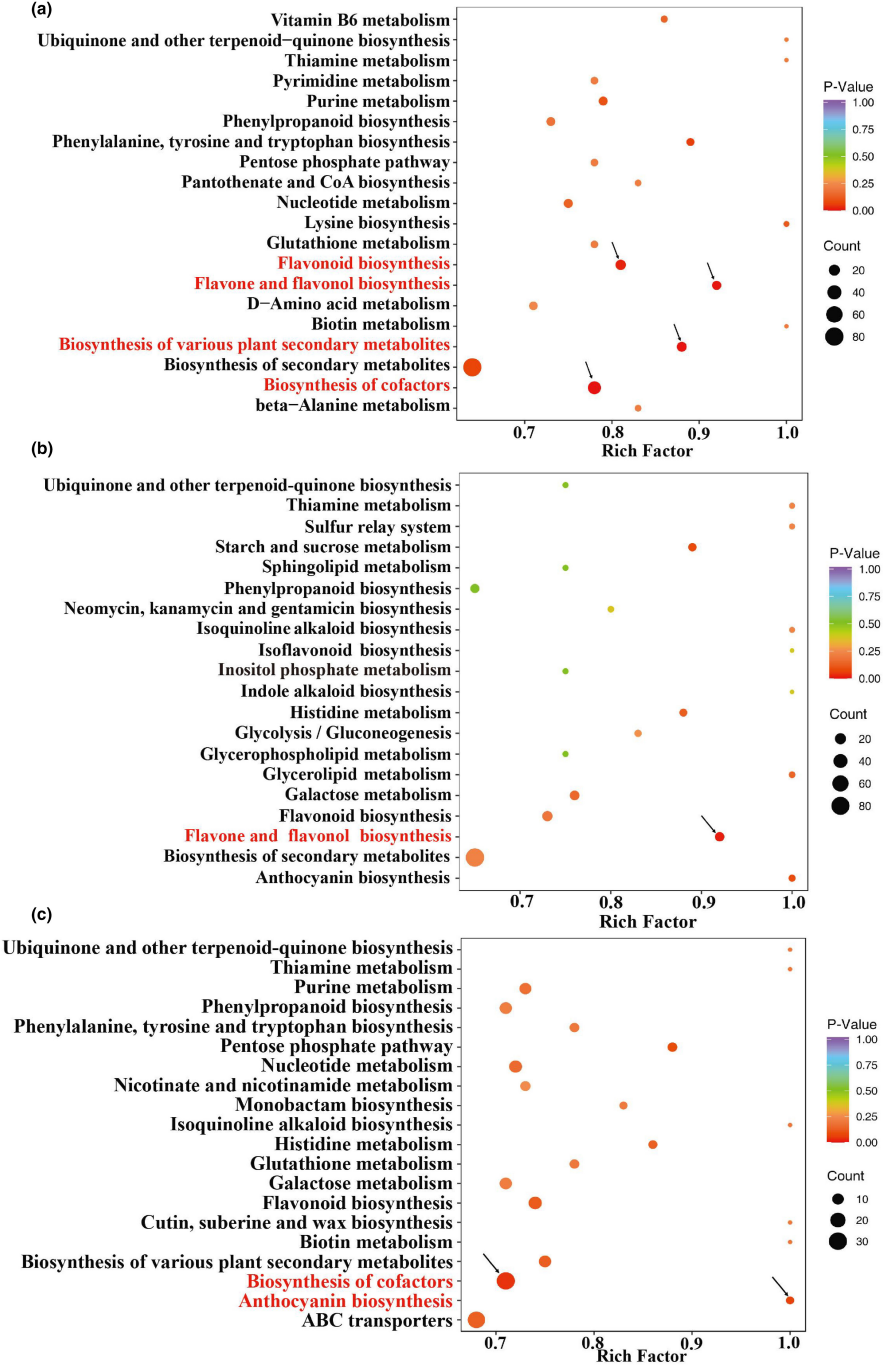
KEGG pathway analysis of differential metabolites between different groups. (a) The pathways that were enriched between peel and aril. (b) Between peel and seed. (c) Between seed and aril.

Enrichment analysis showed that metabolites from the biosynthesis of cofactors (ko01240), flavonoids (ko00941), and flavones and flavonols (ko00944) were significantly enriched between the peel and aril (Figure 5a). The metabolites from flavone and flavonol biosynthesis (ko00944) differed significantly between the peels and seeds (Figure 5b). Metabolic pathways related to the biosynthesis of cofactors (ko01240) and anthocyanins (ko00942) were significantly enriched between seeds and arils (Figure 5c).

### 3.3 Distribution of carbohydrates, organic acids, and amino acids in pomegranate peel, aril, and seed

**FIGURE 6:**
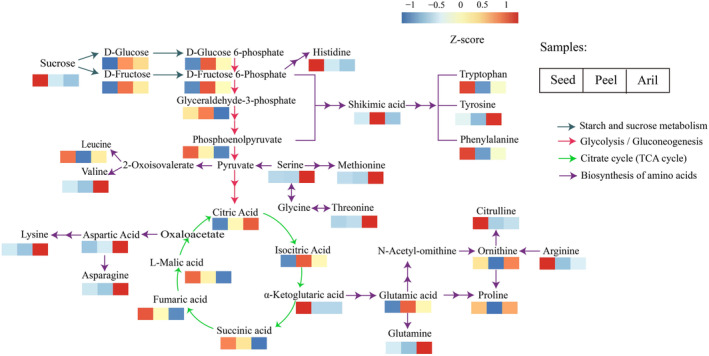
Heatmap of mean normalized carbohydrates, organic acids, and amino acids relative concentrations in pomegranate seed, peel, and aril.

Carbohydrates, organic acids, and amino acids are the major primary metabolites that determine the flavor and nutritional value of pomegranate fruits (Bar‐Ya'akov et al., 2019). Mainly 19 sugars, including D‐glucose, D‐fructose, melibiose, D‐mannose, D‐sorbitol, D‐sucrose, D‐trehalose, etc., were identified (Table S1), of which D‐fructose, D‐glucose, and D‐mannose were upregulated in the peel and aril compared with seeds, while sucrose, dulcitol, D‐mannitol, and D‐sorbitol were mainly upregulated in the seed (Figure 6, Table S1). Moreover, 18 main amino acids were detected in the pomegranate fruit, of which L‐serine, L‐proline, L‐valine, L‐threonine, and L‐leucine showed no significant differences among the groups (Figure 6). L‐asparagine, L‐aspartic acid, L‐glutamine, L‐lysine, and L‐methionine were upregulated in the arils (Figure 6). L‐histidine, L‐arginine, L‐citrulline, L‐tryptophan, and L‐phenylalanine were upregulated in the seeds (Figure 6). L‐Glutamic acid was upregulated in the peel (Figure 6). L‐Ornithine and L‐tyrosine were mainly upregulated in the seeds and arils (Figure 6).

A total of 68 organic acids were identified, including citric acid, isocitric acid, succinic acid, fumaric acid, shikimic acid, and L‐malic acid (Figure 6, Table S1). The contents of shikimic and quinic acid were upregulated in the peel compared with those in the seeds and arils (Figure 6, Table S2–S4). Additionally, α‐ketoglutaric acid was upregulated more in seed than in peel and aril (Figure 6, Table S2–S4). Citric acid, isocitric acid, succinic acid, fumaric acid, and L‐malic acid were identified in all groups of pomegranate fruits, and no significant differences were observed among the different tissues (Figure 6, Tables S2–S4).

To further explore the main factors determining the acidity of pomegranate aril, we performed HPLC to determine the contents of citric acid, malic acid, shikimic acid, oxalic acid, and quininic acid in the arils of the two acidless and three acid varieties. The results showed that citric acid had a decisive effect on pomegranate acidity (Table S6), which is consistent with previous reports (Dafny‐Yalin et al., 2010).

### 3.4 Main functional phenolic acids identified in pomegranate fruit

Phenolic acids are characterized as dietary polyphenols and natural antioxidants that play important roles in biological and pharmacological properties, such as food additives, signaling molecules, anti‐inflammatory, and anticancer (Kumar & Goel, 2019). They are generally present in bound compounds, such as amides, esters, or glycosides (Pereira et al., 2009). Ferulic, caffeic, p‐coumaric, and sinapic acids were the most common phenolic acids (Khoddami et al., 2014). In this study, 196 phenolic acids were identified, of which 120 phenolic acids, including p‐coumaric acid‐4‐O‐glucoside, which had a moderate inhibitory effect on rat liver microsomes CYP3A4 (Ma et al., 2023), and brevifolin carboxylic acid, which moderated antibacterial activity (N'Guessan et al., 2007), were detected in all groups (Figure 5d, Table S1).

**FIGURE 7:**
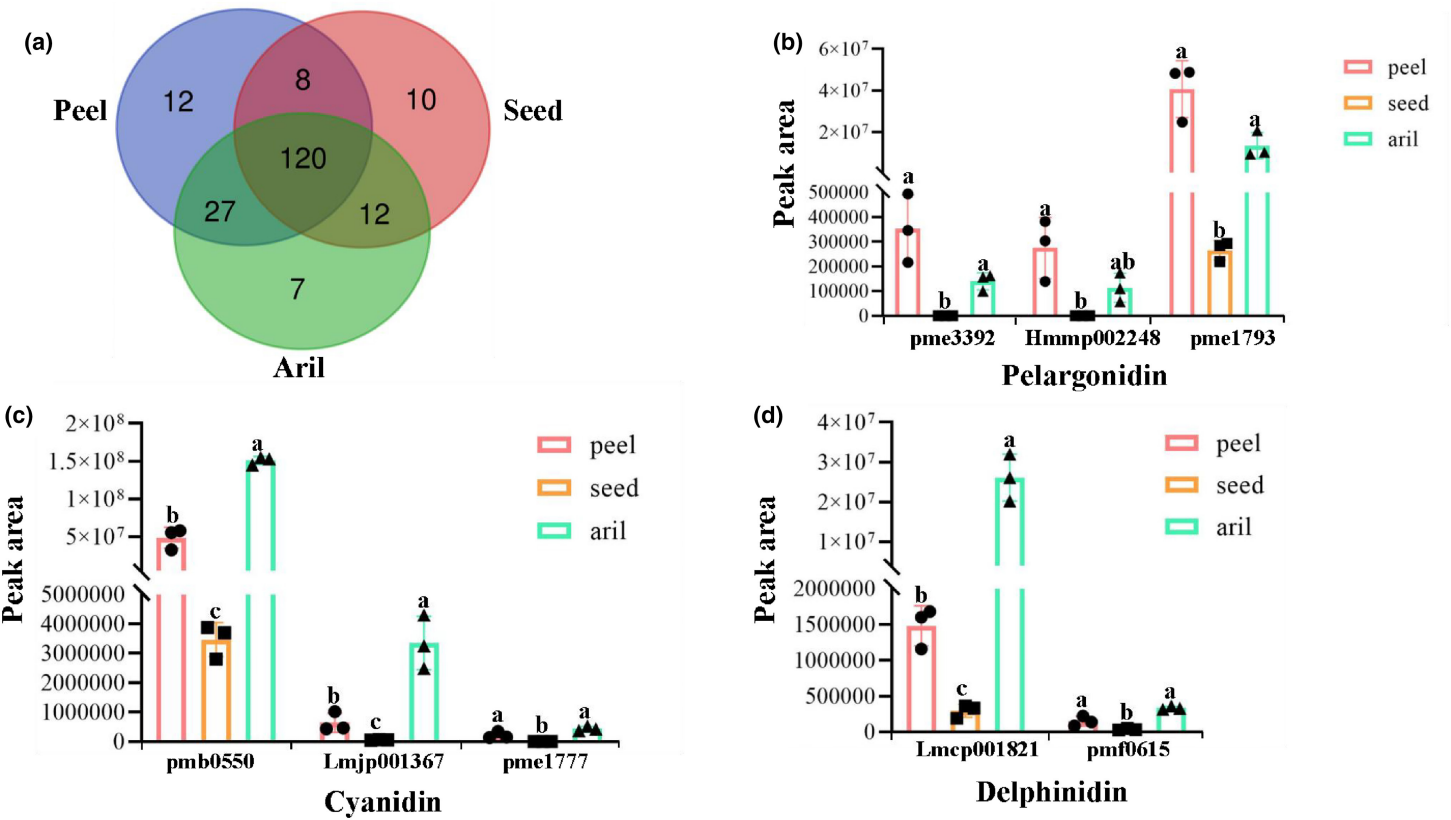
Statistics of differential organic acids and anthocyanidins in pomegranate fruit. (a) The distribution of phenolic acids in pomegranate fruit. (b) The mean and SD values for the peak area of pelargonidin, pme3392, Hmmp002248, and pme1793 were the index of pelargonidin‐3‐O‐glucoside, pelargonidin‐3‐O‐glucoside‐5‐O‐arabinoside, and pelargonidin‐3,5‐O‐diglucoside, respectively. (c) The mean and SD values for the peak area of cyanidin, pmb0550, Lmjp001367, and pme1777 were the index of cyanidin‐3‐O‐glucoside, cyanidin‐3‐O‐(2″‐O‐xylosyl) galactoside, and cyanidin‐3,5‐O‐diglucoside, respectively. (d) The mean and SD values for the peak areas of delphinidin and petunidin. Lmcp001821 and pmf0615 were the indexes of delphinidin‐3‐O‐glucoside and petunidin‐3,5‐di‐O‐glucoside, respectively.

Twelve phenolic acids, such as isochlorogenic acid B/C, which possess various biological properties, including antioxidative, anti‐neurodegenerative, hepatoprotective, and DNA‐protective effects (Liu et al., 2019), were mainly distributed in the aril and seeds. Primarily, 27 members, such as 5‐galloylshikimic acids, 1,4,6‐tri‐O‐galloyl‐β‐D‐glucose, and 3‐galloylshikimic acid, were only obtained in peel and aril. A subset of 12 members, including 1,6‐di‐O‐galloyl‐β‐D‐glucose, which is effective in inhibiting the progress of atherosclerosis (Guo et al., 2007), were identified only in peel (Figure 7a, Table S1). Moreover, 10 members, including p‐coumaric acid, possess biological activities, including antioxidant, anti‐cancer, and antimicrobial (Pei et al., 2016). Coniferaldehyde, which modulates cell wall lignin structure (Takeda et al., 2017), and vanillic acids, which have protective effects against diabetes and diabetic nephropathy (Singh et al., 2022), were only detected in the seeds (Figure 7a, Table S1). A total of seven phenolic acids, including 4‐O‐(6’‐O‐glucosyl‐p‐coumaroyl)‐4‐hydroxybenzyl alcohol, trigallic acid, and 1,7‐Di‐O‐galloyl‐D‐sedoheptulose, were only detected in aril (Figure 7a, Table S1).

### 3.5 Distribution of anthocyanins, punicalagin, and ellagic acid in pomegranate peel, aril, and seed

Anthocyanidins are one of the main flavonoids that contribute to the color of plant flowers and fruits, have antioxidant and health‐promoting properties, and protect the plant from adverse effects of the environment (Zhao & Yuan, 2021). Various anthocyanins have been identified in pomegranates. Pelargonidin‐3‐O‐glucoside, pelargonidin‐3,5‐O‐diglucoside, cyanidin‐3‐O‐glucoside, cyanidin‐3,5‐O‐diglucoside, delphinidin‐3‐O‐glucoside, and petunidin‐3,5‐di‐O‐glucoside are the six major anthocyanins found in pomegranate aril and peel (Zhao & Yuan, 2021). In this study, eight anthocyanidins were detected, including three pelargonidins (pelargonidin‐3‐O‐glucoside, pelargonidin‐3‐O‐glucoside‐5‐O‐arabinoside, and pelargonidin‐3,5‐O‐diglucoside), three cyanidins (cyanidin‐3‐O‐glucoside, cyanidin‐3‐O‐(2″‐O‐xylosyl) galactoside, and cyanidin‐3,5‐O‐diglucoside), delphinidin‐3‐O‐glucoside, and petunidin‐3,5‐di‐O‐glucoside (Figure 7b–d, Table S1). We analyzed the distribution of six major anthocyanins. Cyanidin‐3‐O‐glucoside (Figure 7c) and delphinidin‐3‐O‐glucoside (Figure 7d) contents were significantly higher in the aril than in the peel or seed. Pelargonidin‐3‐O‐glucoside and pelargonidin‐3‐O‐glucoside‐5‐O‐arabinoside were mainly identified in the peel and aril, but no significant differences were observed between them (Figure 7b). Moreover, the contents of pelargonidin‐3,5‐O‐diglucoside (Figure 7b), cyanidin‐3,5‐O‐diglucoside (Figure 7c), and petunidin‐3,5‐di‐O‐glucoside (Figure 7d) were higher in the peel and aril than those in the seed. Of these, cyanidin‐3‐O‐(2″‐O‐xylosyl) galactoside, which may be useful as a novel chemopreventive agent by inhibiting UVB‐induced JNK, MKK3/6, and MEK/ERK1/2 phosphorylation (Jung et al., 2013), was upregulated in the aril compared to that in the peel and seed (Figure 7). Pelargonidin‐3‐O‐glucoside‐5‐O‐arabinoside was mainly identified in peels and arils (Figure 7).

Punicalagin and ellagic acid are the major groups of hydrolyzable tannins in pomegranates, which are responsible for their antioxidant activities (Bar‐Ya'akov et al., 2019; Qin et al., 2020; Tzulker et al., 2007). Our results showed that the level of punicalagin was higher in the peel than that in the aril, whereas no punicalagin was detected in the seeds. Moreover, ellagic acid and its derivatives are among the most attractive functional components owing to their antioxidant and anticancer functions (Maas et al., 1991; Stoner & Morse, 1997; Zheng et al., 2020). Our results showed a high abundance of ellagic acid in different parts of the pomegranate fruit. The ellagic acid content in the peel was significantly higher than that in the arils and seeds. Moreover, we first identified eight ellagic acid derivatives from pomegranate fruit, including 3‐O‐methylellagic acid, 3,3′‐O‐dimethylellagic acid, and 3,3′,4‐O‐trimethylellagic acid, which undergo multiple free radical scavenging processes (Zheng et al., 2020). 3,3′‐O‐dimethylellagic acid and 3,3′,4‐O‐trimethylellagic acid were only identified from peel and seed, while 3‐O‐methylellagic acid was present in all parts of pomegranate fruit (Table S1). Ellagic acid‐4‐O‐rhamnoside was detected only in the peel and arils (Table S1).

### 3.6 Pomegranate seeds were rich in fatty acids and alkaloids

The physicochemical properties and fatty acid profiles of pomegranate seeds have been analyzed in many reports (Fadavi et al., 2006; Khoddami et al., 2013; Özcan et al., 2020). Eight fatty acids, including palmitic acid (C16:0), stearic acid (C18:0), oleic acid (C18:1), linoleic acid (C18:2), and isomers of linolenic acid (C18:3), were obtained from pomegranate seeds, of which punicic acid (C18:3) constituted the highest percentage (~70%) (Fadavi et al., 2006; Khoddami et al., 2014). Here, we found 63 fatty acids abundant in pomegranate seed, mainly including C18:3 class (punicic acid, α‐linolenic acid, and 9‐Hydroxy‐12‐oxo‐10(E),15(Z)‐octadecadienoic acid), C18:2 class (such as linoleic acid, 13(S)‐hydroxyoctadeca‐9Z,11E‐dienoic acid, and 9S‐hydroxy‐10E,12Z‐octadecadienoic acid), C18:1 class (petroselinic acid, elaidic acid, 11‐octadecanoic acid, and hydroxy ricinoleic acid), C18:0 class (stearic acid), palmitic acid (C16:0), and myristic acid (C14:0). Some fatty acids with bioactive or self‐defense properties against plant diseases, such as 13(S)‐Hydroxyoctadeca‐9Z,11E‐dienoic acid, and 9S‐Hydroxy‐10E,12Z‐octadecadienoic acid, were first identified in pomegranate fruits.

Additionally, alkaloids were known to exhibit useful bioactivities; many alkaloids were highly enriched in pomegranate seed, such as indole (Chripkova et al., 2016) and choline (Kansakar et al., 2023), which exhibit significant anticancer, chemopreventive, and antiproliferative activities.

## 4 CONCLUSION

In this study, we successfully performed widely targeted UPLC–MS/MS‐based metabolite profiling of different pomegranate fruit tissues. A total of 858 metabolites were identified, providing comprehensive information on both metabolite composition and abundance in pomegranate fruit. The distributions of carbohydrates, organic acids, amino acids, phenolics, flavonoids, tannins, terpenoids, alkaloids, and fatty acids were analyzed. Our results may lay the foundation for further investigations of the nutritional and medicinal properties of pomegranates.

## AUTHOR CONTRIBUTIONS

**Lina Chen:** Conceptualization (equal); data curation (equal); formal analysis (equal); funding acquisition (equal); investigation (equal); methodology (equal); project administration (equal); resources (equal); software (equal); supervision (equal); validation (equal); visualization (equal); writing – original draft (equal); writing – review and editing (equal). **Ruitao Liu:** Formal analysis (supporting); validation (supporting). **Juanli Zhu:** Investigation (supporting). **Luwei Wang:** Software (supporting); validation (supporting). **Haoxian Li:** Resources (supporting). **Junhao Liu:** Writing – review and editing (supporting). **Zhenhua Lu:** Conceptualization (equal); data curation (equal); project administration (equal); resources (equal); software (equal); supervision (equal); validation (equal); writing – review and editing (equal).

## CONFLICT OF INTEREST STATEMENT

The authors declare that they have no competing financial interests or personal relationships that may have influenced the work reported in this study.

## Supporting information


Supplementary Table S1.

Supplementary Table S2.

Supplementary Table S3.

Supplementary Table S4.

Supplementary Table S5.

Supplementary Table S6.

## Data Availability

The composition and relative content of metabolites identified in the study have been deposited in the figshare database at https://doi.org/10.6084/m9.figshare.25680162.v1.

## References

[fsn34264-bib-0001] Abid, M. , Yaich, H. , Cheikhrouhou, S. , Khemakhem, I. , Bouaziz, M. , Attia, H. , & Ayadi, M. A. (2017). Antioxidant properties and phenolic profile characterization by LC‐MS/MS of selected Tunisian pomegranate peels. Journal of Food Science and Technology, 54, 2890–2901. 10.1007/s13197-017-2727-0 28928529 PMC5583119

[fsn34264-bib-0002] Bar‐Ya'akov, I. , Tian, L. , Amir, R. , & Holland, D. (2019). Primary metabolites, anthocyanins, and hydrolyzable tannins in the pomegranate fruit. Frontiers in Plant Science, 10, 620. 10.3389/fpls.2019.00620 31164897 PMC6534183

[fsn34264-bib-0003] Borochov‐Neori, H. , Judeinstein, S. , Harari, M. , Bar‐Ya'akov, I. , Patil, B. S. , Lurie, S. , & Holland, D. (2011). Climate effects on anthocyanin accumulation and composition in the pomegranate (*Punica granatum* L.) fruit arils. Journal of Agricultural and Food Chemistry, 59, 5325–5334. 10.1021/jf2003688 21506517

[fsn34264-bib-0004] Chen, W. , Gong, L. , Guo, Z. , Wang, W. S. , Zhang, H. Y. , Liu, X. Q. , Yu, S. B. , Xiong, L. Z. , & Luo, J. (2013). A novel integrated method for large‐scale detection, identification, and quantification of widely targeted metabolites: Application in the study of rice metabolomics. Molecular Plant, 6(6), 1769–1780. 10.1093/mp/sst080 23702596

[fsn34264-bib-0005] Chen, Y. , Zhang, R. , Song, Y. , He, J. , Sun, J. , Bai, J. , An, Z. , Dong, L. , Zhan, Q. , & Abliz, Z. (2009). RRLC‐MS/MS‐based metabonomics combined with in‐depth analysis of metabolic correlation network: Finding potential biomarkers for breast cancer. Analyst, 134(10), 2003–2011. 10.1039/B907243H 19768207

[fsn34264-bib-0006] Cheshomi, H. , Bahrami, A. R. , Rafatpanah, H. , & Matin, M. M. (2022). The effects of ellagic acid and other pomegranate (*Punica granatum* L.) derivatives on human gastric cancer AGS cells. Human & Experimental Toxicology, 41, 1–16. 10.1177/09603271211064534 35179410

[fsn34264-bib-0007] Chong, J. , & Xia, J. (2018). MetaboAnalystR: An R package for flexible and reproducible analysis of metabolomics data. Bioinformatics, 34(24), 4313–4314. 10.1093/bioinformatics/bty528 29955821 PMC6289126

[fsn34264-bib-0008] Chripkova, M. , Zigo, F. , & Mojzis, J. (2016). Antiproliferative effect of indole phytoalexins. Molecules, 21(12), 1626. 10.3390/molecules21121626 27898039 PMC6274154

[fsn34264-bib-0009] Cui, B. , Liu, S. M. , & Zheng, T. (2022). Chemotaxonomic identification of key taste and nutritional components in “Shushanggan apricot” fruits by widely targeted metabolomics. Molecules, 27(12), 3870. 10.3390/molecules27123870 35744991 PMC9227342

[fsn34264-bib-0010] Dafny‐Yalin, M. , Glazer, I. , Bar‐Ilan, I. , Kerem, Z. , Holland, D. , & Amir, R. (2010). Color, sugars and organic acids composition in aril juices and peel homogenates prepared from different pomegranate accessions. Journal of Agricultural and Food Chemistry, 58(7), 4342–4352. 10.1021/jf904337t 20232916

[fsn34264-bib-0011] Elfalleh, W. , Tlili, R. , Ying, M. , Sheng‐Hua, H. , Ferchichi, A. , & Nasri, N. (2011). Organoleptic quality, minerals, proteins and amino acids from two Tunisian commercial pomegranate fruits. International Journal of Food Engineering, 7, 12. 10.2202/1556-3758.2057

[fsn34264-bib-0012] Eriksson, L. , Johansson, E. , Kettaneh‐Wold, N. , Trygg, J. , Wikström, C. , & Wold, S. (2006). Multi‐ and megavariate data analysis: Part I: Basic principles and applications (pp. 1–103). MKS Umetrics AB. 10.1201/b14117-9

[fsn34264-bib-0013] Fadavi, A. , Barzegar, M. , & Azizi, M. H. (2006). Determination of fatty acids and total lipid content in oilseed of 25 pomegranates varieties grown in Iran. Journal of Food Composition and Analysis, 19, 676–680. 10.1016/j.jfca.2004.09.002

[fsn34264-bib-0014] Feng, L. J. , Wang, C. Z. , Yang, X. M. , Jiao, Q. Q. , & Yin, Y. L. (2022). Transcriptomics and metabolomics analyses identified key genes associated with sugar and acid metabolism in sweet and sour pomegranate cultivars during the developmental period. Plant Physiology and Biochemistry, 181, 12–22. 10.1016/j.plaphy.2022.04.007 35421745

[fsn34264-bib-0015] Fraga, C. G. , Clowers, B. H. , Moore, R. J. , & Zink, E. M. (2010). Signature‐discovery approach for sample matching of a nerve‐agent precursor using liquid chromatography‐mass spectrometry, XCMS, and chemometrics. Analytical Chemistry, 82(10), 4165–4173. 10.1021/ac1003568 20405949

[fsn34264-bib-0016] Gosset‐Erard, C. , Zhao, M. , Lordel‐Madeleine, S. , & Ennahar, S. (2021). Identification of punicalagin as the bioactive compound behind the antimicrobial activity of pomegranate (*Punica granatum* L.) peels. Food Chemistry, 352, 129396. 10.1016/j.foodchem.2021.129396 33652195

[fsn34264-bib-0017] Guo, A. X. , Huang, X. G. , & Tang, X. Y. (2007). Mechanism of anti‐atherogenesis effect of soluble tannins from *Fructus Phyllanthi* . Practical Preventive Medicine, 14(2), 352–355.

[fsn34264-bib-0018] Hasnaoui, N. , Mars, M. , Ghaffari, S. , Trifi, M. , Melgarejo, P. , & Hernandez, F. (2011). Seed and juice characterization of pomegranate fruits grown in Tunisia: Comparison between sour and sweet cultivars revealed interesting properties for prospective industrial applications. Industrial Crops and Products, 33, 374–381. 10.1016/j.indcrop.2010.11.006

[fsn34264-bib-0019] Hasnaoui, N. , Wathelet, B. , & Jiménez‐Araujo, A. (2014). Valorization of pomegranate peel from 12 cultivars: Dietary fiber composition, antioxidant capacity and functional properties. Food Chemistry, 160, 196–203. 10.1016/j.foodchem.2014.03.089 24799227

[fsn34264-bib-0020] Holland, D. , Hatib, K. , & Bar‐Yaakov, I. (2009). Pomegranate: Botany, horticulture, breeding. Horticultural Reviews, 35, 127–191. 10.1002/9780470593776.ch2

[fsn34264-bib-0021] Jung, S. K. , Lim, T. G. , Seo, S. G. , Lee, H. G. , Hwang, Y. S. , Choung, M. G. , & Lee, K. W. (2013). Cyanidin‐3‐O‐(2″‐xylosyl)‐glucoside, an anthocyanin from Siberian ginseng (*Acanthopanax senticosus*) fruits, inhibits UVB‐induced COX‐2 expression and AP‐1 transactivation. Food Science and Biotechnology, 22, 507–513. 10.1007/s10068-013-0108-7

[fsn34264-bib-0022] Kalaycıoğlu, Z. , & Erim, F. B. (2017). Total phenolic contents, antioxidant activities, and bioactive ingredients of juices from pomegranate cultivars worldwide. Food Chemistry, 221, 496–507. 10.1016/j.foodchem.2016.10.084 27979233

[fsn34264-bib-0023] Kanehisa, M. , & Goto, S. (2000). KEGG: Kyoto encyclopedia of genes and genomes. Nucleic Acids Research, 28(1), 27–30. 10.1093/nar/28.1.27 10592173 PMC102409

[fsn34264-bib-0024] Kansakar, U. , Trimarco, V. , Mone, P. , Varzideh, F. , Lombardi, A. , & Santulli, G. (2023). Choline supplements: An update. Frontiers in Endocrinology (Lausanne), 14, 1148166. 10.3389/fendo.2023.1148166 PMC10025538 36950691

[fsn34264-bib-0025] Khoddami, A. , Man, Y. B. C. , & Roberts, T. H. (2014). Physico‐chemical properties and fatty acid profile of seed oils from pomegranate (*Punica granatum* L.) extracted by cold pressing. European Journal of Lipid Science and Technology, 116(5), 553–562. 10.1002/ejlt.201300416

[fsn34264-bib-0026] Khoddami, A. , Wilkes, M. A. , & Roberts, T. H. (2013). Techniques for analysis of plant phenolic compounds. Molecules, 18, 2328–2375. 10.3390/molecules18022328 23429347 PMC6270361

[fsn34264-bib-0027] Khwairakpam, A. D. , Bordoloi, D. , Thakur, K. K. , Monisha, J. , Arfuso, F. , Gautam Sethi, G. , Mishra, S. , Kumar, A. P. , & Kunnumakkara, A. B. (2018). Possible use of *Punica granatum* L (pomegranate) in cancer therapy. Pharmacological Research, 133, 53–64. 10.1016/j.phrs.2018.04.021 29729421

[fsn34264-bib-0028] Kumar, N. , Daniloski, D. , Pratibha , Neeraj , D'Cunha, N. M. , Naumovski, N. , & Petkoska, A. T. (2022). Pomegranate peel extract—A natural bioactive addition to novel active edible packaging. Food Research International, 156, 111378. 10.1016/j.foodres.2022.111378 35650986

[fsn34264-bib-0029] Kumar, N. , & Goel, N. (2019). Phenolic acids: Natural versatile molecules with promising therapeutic applications. Biotechnology Reports, 24, e00370. 10.1016/j.btre.2019.e00370 31516850 PMC6734135

[fsn34264-bib-0030] Larrosa, M. , González‐Sarrías, A. , Yáñez‐Gascón, M. J. , Selma, M. V. , Azorín‐Ortuño, M. , Toti, S. , Tomás‐Barberán, F. , Dolara, P. , & Espín, J. C. (2010). Anti‐inflammatory properties of a pomegranate extract and its metabolite urolithin‐A in a colitis rat model and the effect of colon inflammation on phenolic metabolism. Journal of Nutritional Biochemistry, 21(8), 717–725. 10.1016/j.jnutbio.2009.04.012 19616930

[fsn34264-bib-0031] Li, Y. C. , Gu, P. , Wang, L. W. , Wang, S. Y. , Yang, H. Y. , Zhang, B. L. , Zhu, B. Q. , & Ma, C. (2017). Comparison of amino acid profile in the juice of six pomegranate cultivars from two cultivation regions in China. Journal of Food Processing and Preservation, 41, e13197. 10.1111/jfpp.13197

[fsn34264-bib-0032] Liu, G. M. , Xu, X. , Hao, Q. F. , & Gao, Y. X. (2009). Supercritical CO2 extraction optimization of pomegranate (*Punica granatum* L.) seed oil using response surface methodology. LWT‐Food Science and Technology, 42, 1491–1495. 10.1016/j.lwt.2009.04.011

[fsn34264-bib-0033] Liu, X. , Zhang, B. , Mei, D. , & Huang, K. (2019). Rapid and sensitive HPLC‐MS/MS method for quantitative determination of isochlorogenic acid B in rat plasma and its application in pharmacokinetic study. Journal of Chinese Pharmaceutical Sciences, 28(3), 167–173. 10.5246/jcps.2019.03.016

[fsn34264-bib-0034] Ma, C. Y. , Liu, C. Y. , Ren, M. J. , Cui, L. L. , Xi, X. F. , & Kang, W. Y. (2023). Inhibitory effect of quercetin‐3‐O‐α‐rhamnoside, p‐coumaric acid, phloridzin and 4‐O‐β‐glucopyranosyl‐cis‐coumaric acid on rats liver microsomes cytochrome P450 enzyme activities. Food and Chemical Toxicology, 172, 113583. 10.1016/j.fct.2022.113583 36577462

[fsn34264-bib-0035] Maas, J. L. , Wang, S. Y. , & Galletta, G. J. (1991). Evaluation of strawberry cultivars for ellagic acid content. HortScience, 26(1), 66–68. 10.1016/0304-4238(91)90102-5

[fsn34264-bib-0036] Melgarejo, P. , & Artes, F. (2000). Total lipid content and fatty acid composition of oil seed from lesser known sweet pomegranate clones. Journal of the Science of Food and Agriculture, 80, 1452–1454. 10.1002/1097-0010(200008)80:103.0.CO;2-L

[fsn34264-bib-0037] Melgarejo, P. , Salazar, D. , & Artes, F. (2000). Organic acids and sugars composition of harvested pomegranate fruits. European Food Research and Technology, 211, 185–190. 10.1007/s002170050021

[fsn34264-bib-0038] Mphahlele, R. R. , Caleb, O. J. , Fawole, O. A. , & Opara, U. L. (2016). Effects of different maturity stages and growing locations on changes in chemical, biochemical and aroma volatile composition of ’Wonderful’ pomegranate juice. Journal of the Science of Food and Agriculture, 96, 1002–1009. 10.1002/jsfa.7186 25809070

[fsn34264-bib-0039] N'Guessan, J. D. , Bidie, A. P. , Lenta, B. N. , Weniger, B. , Andre, P. , & Guede‐Guina, F. (2007). In vitro assays for bioactivity‐guided isolation of antisalmonella and antioxidant compounds in Thonningia sanguinea flowers. African Journal of Biotechnology, 14, 1685–1689. 10.1186/1471-2105-8-260

[fsn34264-bib-0040] Özcan, M. M. , Alkaltham, M. S. , Uslu, N. , & Salamatullah, A. (2020). Effect of different roasting methods on the bioactive properties, phenolic compounds and fatty acid compositions of pomegranate (*Punica granatum* L. cv. Hicaz) seed and oils. Journal of Food Science and Technology, 58(6), 2283–2294. 10.1007/s13197-020-04739-1 33967325 PMC8076372

[fsn34264-bib-0041] Pei, K. , Ou, J. Y. , Huang, J. Q. , & Ou, S. Y. (2016). p‐Coumaric acid and its conjugates: Dietary sources, pharmacokinetic properties and biological activities. Journal of the Science of Food and Agriculture, 96(9), 2952–2962. 10.1002/jsfa.7578 26692250

[fsn34264-bib-0042] Pereira, D. M. , Valentão, P. , Pereira, J. A. , & Andrade, P. B. (2009). Phenolics: From chemistry to biology. Molecules, 14(6), 2202–2211. 10.3390/molecules14062202

[fsn34264-bib-0043] Poyrazolua, E. , Gokmen, V. , & Artik, N. (2002). Organic acids and phenolic compounds in pomegranates (*Punica granatum* L.) grown in Turkey. Journal of Food Composition and Analysis, 15, 567–575. 10.1006/jfca.2002.1071

[fsn34264-bib-0044] Qin, G. H. , Liu, C. Y. , Li, J. Y. , Gao, Z. , Qi, Y. J. , Zhang, X. H. , Yi, X. K. , Pan, H. F. , Ming, R. , & Xu, Y. L. (2020). Diversity of metabolite accumulation patterns in inner and outer seed coats of pomegranate: Exploring their relationship with genetic mechanisms of seed coat development. Horticulture Research, 7, 10. 10.1038/s41438-019-0233-4 31934341 PMC6946660

[fsn34264-bib-0045] Rowayshed, G. , Salama, A. , Abul‐Fadl, M. , Akila‐Hamza, S. , & Mohamed, E. A. (2013). Nutritional and chemical evaluation for pomegranate (*Punica granatum* L.) fruit peel and seeds powders by products. Middle East Journal of Applied Science, 3(4), 169–179.

[fsn34264-bib-0046] Sharma, P. , McClees, S. F. , & Afaq, F. (2017). Pomegranate for prevention and treatment of cancer: An update. Molecules, 22(1), 177. 10.3390/molecules22010177 28125044 PMC5560105

[fsn34264-bib-0047] Singh, B. , Kumar, A. , Singh, H. , Kaur, S. , Arora, S. , & Singh, B. (2022). Protective effect of vanillic acid against diabetes and diabetic nephropathy by attenuating oxidative stress and upregulation of NF‐kappa B, TNF‐alpha and COX‐2 proteins in rats. Phytotherapy Research, 36(3), 1338–1352. 10.1002/ptr.7392 35088468

[fsn34264-bib-0048] Stoner, G. D. , & Morse, M. A. (1997). Isothiocyanates and plant polyphenols as inhibitors of lung and esophageal cancer. Cancer Letter, 114(1–2), 113–119. 10.1016/s0304-3835(97)04639-9 9103268

[fsn34264-bib-0049] Takeda, Y. , Koshiba, T. , Tobimatsu, Y. , Suzuki, S. , Murakami, S. , Yamamura, M. , Rahman, M. M. , Takano, T. , Hattori, T. , Sakamoto, M. , & Umezawa, T. (2017). Regulation of *CONIFERALDEHYDE 5‐HYDROXYLASE* expression to modulate cell wall lignin structure in rice. Planta, 246(2), 337–349. 10.1007/s00425-017-2692-x 28421330

[fsn34264-bib-0050] Thévenot, E. A. , Roux, A. , Xu, Y. , Ezan, E. , & Junot, C. (2015). Analysis of the human adult urinary metabolome variations with age, body mass index, and gender by implementing a comprehensive workflow for univariate and opls statistical analyses. Journal of Proteome Research, 14(8), 3322–3335. 10.1021/acs.jproteome.5b00354 26088811

[fsn34264-bib-0051] Turrini, E. , Ferruzzi, L. , & Fimognari, C. (2015). Potential effects of pomegranate polyphenols in cancer prevention and therapy. Oxidative Medicine and Cellular Longevity, 2015, 938475. 10.1155/2015/938475 26180600 PMC4477247

[fsn34264-bib-0052] Tzulker, R. , Glazer, I. , Bar‐Ilan, I. , Holland, D. , Aviram, M. , & Amir, R. (2007). Antioxidant activity, polyphenol content, and related compounds in different fruit juices and homogenates prepared from 29 different pomegranate accessions. Journal of Agricultural and Food Chemistry, 55(23), 9559–9570. 10.1021/jf071413n 17914875

[fsn34264-bib-0053] Vicinanza, R. , Zhang, Y. , Henning, S. M. , & Heber, D. (2013). Pomegranate juice metabolites, ellagic acid and urolithin A, synergistically inhibit androgen‐independent prostate cancer cell growth via distinct effects on cell cycle control and apoptosis. Evidence‐Based Complementary and Alternative Medicine, 2013, 247504. 10.1155/2013/247504 23710216 PMC3655614

[fsn34264-bib-0054] Viuda‐Martos, M. , Fernández‐López, J. , & Pérez‐álvarez, J. A. (2010). Pomegranate and its many functional components as related to human health: A review. Comprehensive Reviews in Food Science & Food Safety, 9(6), 635–654. 10.1111/j.1541-4337.2010.00131.x 33467822

[fsn34264-bib-0055] Yang, J. , Guo, Y. , Lee, R. , Henning, S. M. , Wang, J. , Pan, Y. , Qing, T. , Hsu, M. , Nguyen, A. , Prabha, S. , Ojha, R. , Small, G. W. , Heber, D. , & Li, Z. (2020). Pomegranate metabolites impact tryptophan metabolism in humans and mice. Current Developments in Nutrition, 4(11), nzaa165. 10.1093/cdn/nzaa165 33274309 PMC7695807

[fsn34264-bib-0056] Yaritz, U. , Schweitzer, R. , Holland, D. , Tian, L. , & Amir, R. (2022). Metabolic profiling of outer fruit peels from 15 accessions of pomegranate (*Punica granatum* L.). Journal of Food Composition and Analysis, 109, 104482. 10.1016/j.jfca.2022.104482

[fsn34264-bib-0057] Yin, Q. C. , Ji, J. B. , Zhang, R. H. , Duan, Z. W. , Xie, H. , Chen, Z. , Hu, F. C. , & Deng, H. (2022). Identification and verification of key taste components in wampee using widely targeted metabolomics. Food Chemistry: X, 13, 100261. 10.1016/j.fochx.2022.100261 35499032 PMC9040002

[fsn34264-bib-0058] Zhao, X. Q. , & Yuan, Z. H. (2021). Anthocyanins from pomegranate (*Punica granatum* L.) and their role in antioxidant capacities in vitro. Chemistry Biodiversity, 18(10), e2100399. 10.1002/cbdv.202100399 34388293

[fsn34264-bib-0059] Zhao, X. Q. , Yuan, Z. H. , Fang, Y. M. , Yin, Y. L. , & Feng, L. J. (2013). Characterization and evaluation of major anthocyanins in pomegranate (*Punica granatum* L.) peel of different cultivars and their development phases. European Food Research and Technology, 236, 109–117. 10.1007/s00217-012-1869-6

[fsn34264-bib-0060] Zheng, P. F. , Zhang, M. , Fang, X. , Tang, L. L. , Wang, Z. X. , & Shi, F. C. (2022). Analysis of the fruit quality of pear (*pyrus* spp.) using widely targeted metabolomics. Food, 11, 1440. 10.3390/foods11101440 PMC9140454 35627008

[fsn34264-bib-0061] Zheng, Y. Z. , Fu, Z. M. , Deng, G. , Guo, R. , & Chen, D. F. (2020). Free radical scavenging potency of ellagic acid and its derivatives in multiple H+/e– processes. Phytochemistry, 180, 112517. 10.1016/j.phytochem.2020.112517 32950773

[fsn34264-bib-0062] Zou, S. , Wu, J. , Shahid, M. Q. , He, Y. H. , Lin, S. Q. , Liu, Z. H. , & Yang, X. H. (2020). Identification of key taste components in loquat using widely targeted metabolomics. Food Chemistry, 323, 126822. 10.1016/j.foodchem.2020.126822 32334307

